# Growing-up (habitually) barefoot influences the development of foot and arch morphology in children and adolescents

**DOI:** 10.1038/s41598-017-07868-4

**Published:** 2017-08-14

**Authors:** Karsten Hollander, Johanna Elsabe de Villiers, Susanne Sehner, Karl Wegscheider, Klaus-Michael Braumann, Ranel Venter, Astrid Zech

**Affiliations:** 1 Department of Sports and Exercise Medicine, Institute of Human Movement Science, University of Hamburg, Hamburg, Germany; 2 Department of Sport Science, Stellenbosch University, Stellenbosch, South Africa; 3 Department of Medical Biometry and Epidemiology, University Medical Center Hamburg-Eppendorf, Hamburg, Germany; 4 Department of Sport Science, Friedrich Schiller University Jena, Jena, Germany

## Abstract

The development of the human foot is crucial for motor learning in children and adolescents as it ensures the basic requirements for bipedal locomotion and stable standing. Although there is an ongoing debate of the advantages and disadvantages of early and permanent footwear use, the influence of regular barefootness on foot characteristics in different stages of child development has not been extensively evaluated. A multicenter epidemiological study was conducted to compare the foot morphology between habitually barefoot children and adolescents (N = 810) to age-, sex- and ethnicity-matched counterparts that are used to wearing shoes. While controlling for confounders, we found that habitual footwear use has significant effects on foot-related outcomes in all age groups, such as a reduction in foot arch and hallux angles. The results indicate an impact of habitual footwear use on the development of the feet of children and adolescents. Therefore, growing up barefoot or shod may play an important role for childhood foot development, implying long-term consequences for motor learning and health later in life.

## Introduction

Even though being barefoot has been a part of human evolution for millions of years^1^, over-ground walking and running in industrial countries has been more regularly performed shod in the last few centuries. The advantages and disadvantages of footwear use for (foot) health and the development of motor control are increasingly discussed in the literature^2, 3^ without conclusive findings since long-term effects of being habitually barefoot have rarely been evaluated^4^.

The comparison of shod and unshod feet has been of scientific and clinical interest for more than a century now^5^. Evidence exists that the use of footwear can influence the foot and arch morphology^6–9^. Individuals that have been barefoot almost all their lives (habitually barefoot) seem to have wider feet^4, 8^, as well as fewer foot and toe deformities^4, 9, 10^. These habitually barefoot individuals have a higher foot arch and lower hallux angles compared to habitually shod individuals^4^. This is in accordance with higher rates of differences in foot characteristics like flat feet and hallux valgus deformities in habitually shod populations^9, 11^. Furthermore, it is reported that the pliability of habitually shod feet is reduced compared to habitually barefoot ones^6^.

However, several limitations produce uncertainty regarding the ability to generalize and interpret the reported findings. First, the heterogeneous use of the term “habitually barefoot” questions the comparability of included barefoot populations^4^. Second, data obtained from adult populations^6–8, 10^ cannot be related to child and youth populations. The development of foot and arch characteristics is dependent on several factors, such as body weight^12^, physical activity^13^, ethnicity^14^ and age^15^. Additionally, body weight is associated with a reduced arch height^16^, while children who are more physically active show increased arch heights^13^. Age also exerts influence on foot characteristics. Especially in the first years of life, the arch height of habitually shod children increases, eventually becoming relatively stable after they reach 7 years of age^15^. Therefore, habitual barefootedness may be especially influential during a child’s growing years, leading to the hypothesis that the influence of footwear use may change as long as the feet are growing. Accordingly, a large cohort of habitually barefoot and shod children and adolescents from different age groups is needed in order to investigate if regular shoe use (or regular barefoot locomotion) in the early stages of life influences the anthropometric foot characteristics of children. Such an epidemiological approach should fill the gap in the current research on long-term effects of regular barefoot locomotion.

This study’s main hypothesis was to compare key components of foot characteristics (foot and arch morphology, hallux angles and pliability) between habitually barefoot and habitually shod children and adolescents during different stages of development. With regard to the currently available body of research, we hypothesized that habitually barefoot children would have higher foot arches, reduced hallux angles and increased foot pliability than their shod counterparts.

## Methods

The study reports according to the STROBE guidelines for reporting observational studies^17^.

### Study design

A cross-sectional observational study was conducted in South Africa and Germany between March 2015 and June 2016, the full study protocol from which has been published^18^. Ethical approval has been obtained from the university ethics committee (protocol number HS1153/2014) and the medical association (protocol number PV4971). The study was carried out in accordance with the Helsinki Declaration guidelines. Written informed parental consent and the child’s assent to participate was obtained prior to participation.

### Setting and participants

The data collection was performed in 22 primary and secondary schools across rural and urban areas in the Western Cape and Northern Germany. The regionally separated recruitment was performed due to the obligation to wear footwear at school in Germany, while it is common for South African children to attend school barefoot. After approval from the responsible school authorities, schools in the regions were randomly selected per stratum and contacted by the principle investigators. The response rates of schools in Germany was 22% and in South Africa 55%. In cases of willingness by school directors and physical education teachers to participate, information sheets and consent forms were distributed to all children (and their parents). Volunteers with a signed consent form from the parents were tested at the school during their regular physical education lessons.

Children and adolescents aged 6–18 years who were willing to participate were included when they were healthy and physically active for at least 120 cumulative minutes per week (as reported by their parent(s) or legal guardians(s)). Exclusion criteria consisted of current injuries, as well as orthopaedic, neurological or neuromuscular abnormalities likely to affect the gait (also per parent proxy). For an even recruitment, we aimed to include at least ten female and ten male participants per class level and group.

To determine the independent variable, and due to the lack of standardized definitions in the literature, “habitual barefootness” was tested with a three point Likert scale^18^. Using three items, the children were asked whether they are barefoot most of the time (2 points)/half of the time (1 point)/none of the time (0 points), a) during school, b) during sports and c) in and around the house. Participants were included as habitually barefoot if they had a score of ≥3 (from a maximum 6 points), equivalent to being barefoot at least half of the time at school *or* at sports in addition to being barefoot at home during primary school. The rate of habitually barefoot children in South Africa was 90.9%. In Germany, all the children were habitually shod.

### Data measurement and variables

Prior to the testing period, a joint training of the research teams was held in Germany over several days to ensure the identical use of the equipment and data collection. In addition, part of the German research team (including the researcher leading all testing in Germany) attended the first weeks of testing in South Africa.

The testing protocol for this study consisted of anthropometrical (date of birth, height, weight, foot size), static and dynamic foot measurements. Main foot mechanical measures were seated and standing foot length, foot width and dorsum height, as well as dynamic arch index (dAI) and hallux angle (HA). From this data, static arch height index (sAHI) and pliability ratio (PR) were calculated.

### Static foot measurements

Two specially constructed calipers^18^ were used to measure heel-to-toe length (HTL), foot width (FW) and dorsum height (DH). Both calipers were used in Germany and South Africa and tested for validity. DH was measured at 50% of HTL. Both feet were measured during sitting and standing position. The measured values were used to calculate the static arch height index (sAHI).

1 $${Static}\,{arch}\,{height}\,{index}=\frac{{DH}}{{HTL}}$$

and the pliability ratio according to Kadambande *et al*.^6^:

2 $${Pliability}\,{ratio}=\frac{{HTL}\,50 \% \,{of}\,{BW}\times {FW}\,50 \% \,{of}\,{BW}}{{HTL}\,\mathrm{10} \% \,{of}\,{BW}\times {FW}\,\mathrm{10} \% \,{of}\,{BW}\,}$$

The reliability of this static foot measurement was shown to be good to excellent for children (intraday: 0.88–0.90; inter-rater: 0.80–0.85)^19^.

### Dynamic foot measurements

Dynamic footprints were measured with a capacitance-based pressure platform (Emed n50, Novel GmbH, Munich, Germany) embedded in the middle of a 3 m portable wooden walkway. Using a two-step approach^19, 20^, the mean plantar pressure of three valid walking trials was used for each foot. Participants walked with a comfortable, self-selected speed on the walkway. Only trials in which the foot was fully placed on the pressure plate were used for data analysis. Footprint data were collected and processed with the provided software (Novel database pro m, Version 24.3.20 Novel GmbH, Munich, Germany). The software was used to calculate the dynamic arch index according to Cavanagh and Rodgers^21^ and the hallux angle according to Donatelli and Wolf^22^. A small dynamic arch index corresponds to a high arch, while a high hallux angle corresponds to a valgus deviated hallux. The reliability of dynamic plantar pressure assessment in children has been shown to be excellent (ICC = 0.92) and preferable over static plantar pressure assessments^23^.

### Bias

To address potential sources of bias, we included BMI, ethnicity and the physical activity of each participant as confounding variables. To determine the level of physical activity, we used the validated physical activity questionnaire for children and adolescents (PAQ-C and PAQ-A)^24^.

### Study size

For sample size calculations, we used published values of the dynamic arch index (mean: 0.19, SD: 0.07) from a large cohort^15^. Twenty percent of the average (0.19; i.e., 0.038) was considered to be the minimal important difference. With a significance level of 0.05 and a power of 0.8, we calculated a minimum of 16 participants per age and country to be included.

### Statistical methods

Sample characteristics are given as absolute and relative frequencies or mean+/− standard deviation, whichever is appropriate. All outcome parameters (foot length, foot width, static arch height index, pliability ratio, hallux angle and dynamic arch index) were analysed in separate mixed-effects linear regressions, adjusting for the clustered structure induced by the repeated measurements by side and setting (seated/standing) per child. The predictors habitually barefoot (yes/no) and age (in three different stages of development) and the two-way interaction of both were modelled as fixed effects. In the case of an insignificant interaction term, only the main effects habitually barefoot (yes/no) and age were included. This decision was met by using the likelihood ratio test for model comparison. Moreover, in all models, the following confounders were included: BMI, sex, ethnicity, PAQ-score and side as well as whether the measurement was performed seated or standing, if appropriate. The adjusted results were estimated as marginal means, which are represented in tables and graphs with 95% confidence intervals (95%-CI). Post hoc tests for comparison of the estimated means were calculated with contrast tests using Wald tests. All of the models present available case analyses. For our six main hypotheses, an adjusted alpha using Bonferroni corrections were reported. For all hierarchical hypotheses, nominal p-values were reported without correction for multiplicity. A two-tailed p < 0.05 was considered to be statistically significant. All of the analyses were performed using STATA 14 (StataCorp. 2015. Stata Statistical Software: Release 14. College Station, TX: StataCorp LP).

## Results

### Participants

**Figure 1:**
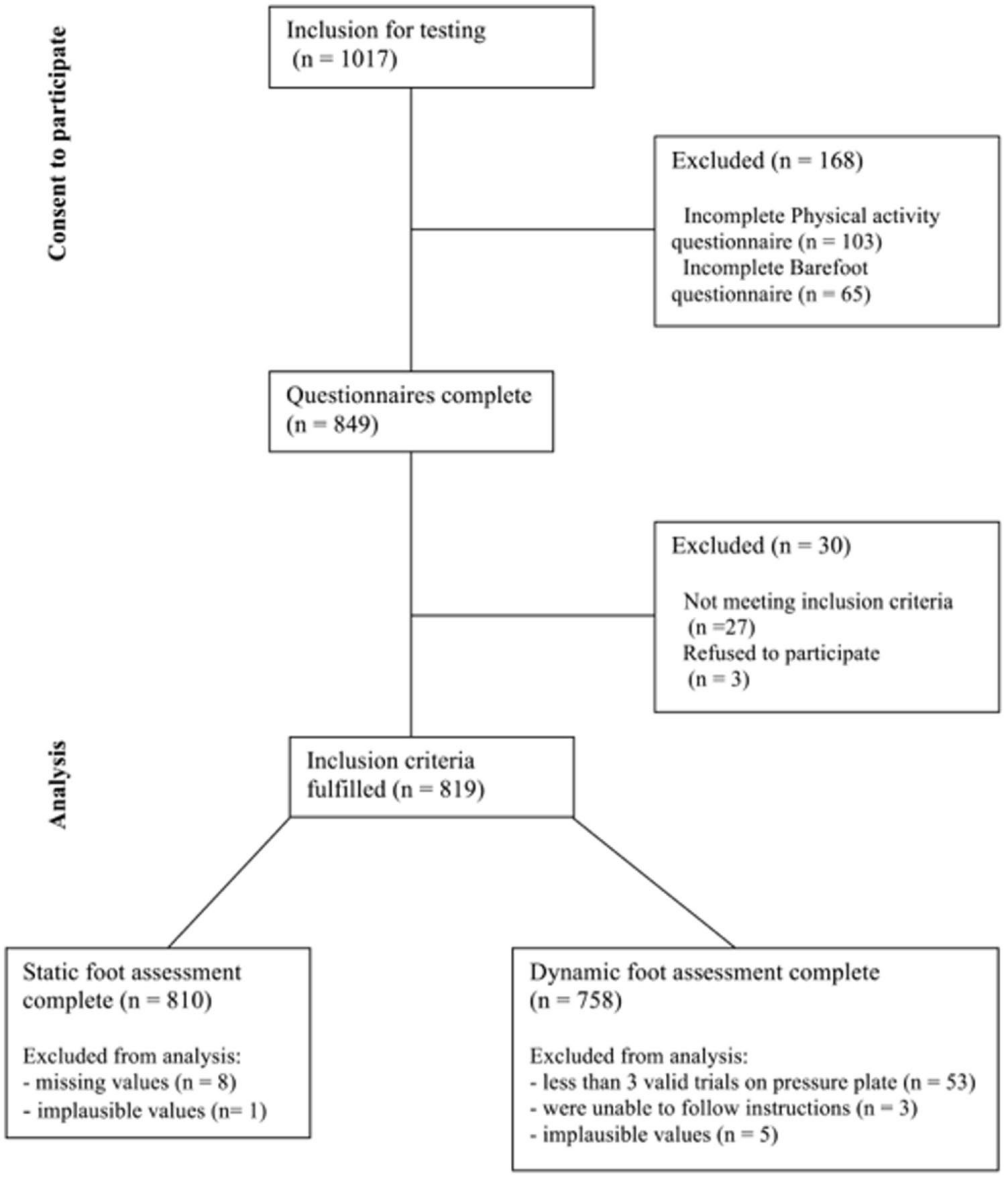
Diagram showing the flow of participants through the study.

Of the initial 1017 children tested, a total of 810 children (50.1% females, 49.9% males) aged 11.99 ± 3.33 years (body height: 153.99 ± 17.91 cm; weight 48.10 ± 17.90; BMI 19.55 ± 3.94; PAQ-Score 2.89 ± 0.68) were included in the analysis. Figure 1 provides an overview of participant flow and reasons for exclusion. All descriptive statistics of the individual subgroups can be found in Table 1.

**Table 1:** Descriptive statistics of all habitually barefoot and habitually shod children included in the analysis.

	Number of participants	Sex	Age [years]	Height [cm]	Weight [kg]	BMI [kg]	PAQ-Score		Foot length [cm]	Foot width [cm]	Static arch height index	Pliability Ratio	Hallux angle [°]	Dynamic arch index
	n	% female	mean (SD)	mean (SD)	mean (SD)	mean (SD)	mean (SD)		mean (SD)	mean (SD)	mean (SD)	mean (SD)	mean (SD)	mean (SD)
**6 to <10 years**														
Habitually shod	101	53.3	8.34 (1.29)	134.70 (9.27)	32.41 (8.67)	17.61 (2.97)	3.20 (0.68)	Sitting	20.23 (1.46)	7.80 (0.53)	0.26 (0.02)	1.06 (0.03)	−0.84 (5.90)	0.15 (0.07)
								Standing	20.62 (1.45)	8.08 (0.51)	0.24 (0.02)			
Habitually barefoot	123	49.2	8.13 (1.24)	134.48 (8.73)	30.45 (7.11)	16.65 (2.40)	2.87 (0.65)	Sitting	20.57 (1.37)	8.00 (0.49)	0.29 (0.02)	1.05 (0.03)	0.69 (5.52)	0.17 (0.08)
								Standing	20.90 (1.38)	8.23 (0.49)	0.27 (0.02)			
**10 to <14 years**														
Habitually shod	155	45.0	12.40 (1.04)	159.18 (10.16)	49.59 (11.81)	19.42 (3.40)	2.78 (0.70)	Sitting	23.42 (1.57)	8.84 (0.59)	0.26 (0.02)	1.05 (0.03)	1.46 (5.55)	0.19 (0.07)
								Standing	23.84 (1.56)	9.11 (0.60)	0.23 (0.02)			
Habitually barefoot	154	45.7	12.41 (1.01)	159.07 (10.11)	52.55 (13.26)	20.55 (3.70)	3.09 (0.63)	Sitting	23.29 (1.58)	8.85 (0.68)	0.28 (0.02) 0.26 (0.02)	1.05 (0.02)	3.32 (5.86)	0.17 (0.07)
								Standing	23.72 (1.59)	9.08 (0.68)				
**14 to 18 years**														
Habitually shod	169	58.20	16.16 (1.09)	171.30 (9.41)	63.67 (14.57)	21.60 (3.98)	2.61 (0.60)	Sitting	24.60 (1.62)	9.30 (0.69)	0.25 (0.02)	1.04 (0.02)	2.90 (6.14)	0.18 (0.07)
								Standing	24.94 (1.65)	9.55 (0.71)	0.23 0.02			
Habitually barefoot	108	48.30	16.06 (0.98)	171.65 (9.67)	67.17 (14.13)	22.68 (3.80)	2.83 (0.60)	Sitting	25.37 (1.95)	9.44 (0.71)	0.28 (0.02)	1.04 (0.02)	4.30 (5.58)	0.20 (0.07)
								Standing	25.76 (1.94)	9.63 (0.71)	0.26 (0.02)			
**Total (6–18 years)**														
Habitually shod	425	50.1	12.40 (3.36)	155.99 (17.53)	48.68 (17.59)	19.33 (3.88)	2.75 (0.66)	Sitting	23.13 (2.32)	8.78 (0.85)	0.26 (0.02)	1.05 (0.03)	1.48 (6.04)	0.18 (0.07)
								Standing	23.51 (2.31)	9.04 (0.85)	0.23 (0.02)			
Habitually barefoot	385	49.9	11.53 (3.24)	151.77 (18.09)	47.47 (18.24)	19.80 (3.99)	3.06 (0.66)	Sitting	23.07 (2.48)	8.77 (0.85)	0.28 (0.02)	1.04 (0.02)	2.76 (5.86)	0.18 (0.07)
								Standing	23.46 (2.50)	8.98 (0.84)	0.26 (0.02)			

### Significant effects of confounders on foot outcome data

**Figure 2:**
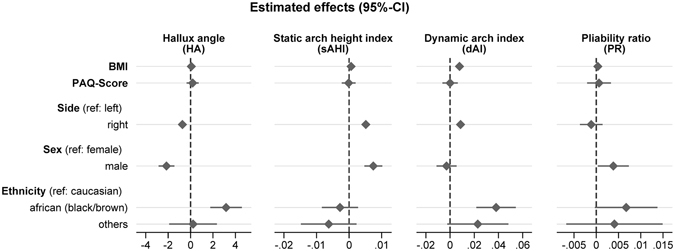
Forrest plots depicting confounder effects estimates for hallux angle, dynamic foot arch index, static arch height index and pliability ratio.

The confounder effect estimates (Fig. 2) show a significant effect for side (p < 0.001), sex (p < 0.001) and BMI (p = 0.002) on static arch height index and for sex (p = 0.031) on pliability ratio. There was no statistically significant effect of ethnicity or physical activity on static foot outcomes. For dynamic foot outcomes, a significant effect of BMI (p < 0.001), side (p < 0.001) and ethnicity (p < 0.001) was observed for the dynamic arch index, while side (p < 0.001), sex (p < 0.001) and ethnicity (p < 0.001) influence the hallux angle. For all significant confounders, the adjusted estimated marginal effects were reported.

### Estimated marginal effects on age-groups

**Figure 3:**
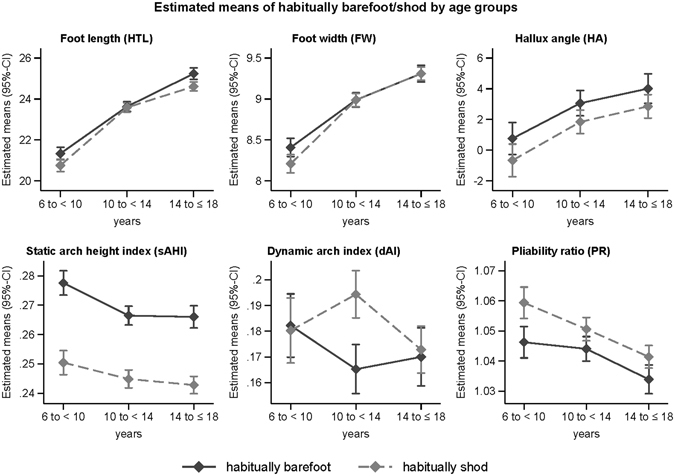
Marginal effects of habitual barefoot *vs*. habitually shod children by age in stages of development showing estimated means and 95%CI for foot length, foot width, hallux angle, static arch height index, dynamic arch index and pliability ratio.

For the dynamic arch index a different development between habitually barefoot and habitually shod individuals can be observed over the three age groups (p_(interaction)_ = 0.004). For all other outcome parameters, the estimated marginal effects show an increase of foot length, foot width and hallux angle with age, while static arch height index and pliability ratio decrease with increasing age (Supplementary Table S1 and Fig. 3).

### Differences between habitually barefoot *vs*. shod children

When comparing habitually barefoot to habitually shod participants, age-independent globally significant differences were found for static arch height index (p < 0.001), pliability ratio (p < 0.001) and hallux angle (p = 0.001) in all participants (Table 2 and Fig. 3). Pairwise comparisons of habitually barefoot and habitually shod participants revealed differences for foot length in age groups 6–10 (p = 0.006) and 14–18 years (p < 0.001), for foot width in age group 6–10 years (p = 0.010) and for dynamic arch index in age group 10–14 years (p < 0.001) (Table 2 and Fig. 3).

**Table 2:** Pairwise comparisons of habitually barefoot/shod children by age in stages of development.

Estimated marginal difference of habitually barefoot *vs*. habitually shod		Foot length [cm]	Foot width [cm]	Static arch height index	Pliability Ratio	Dynamic arch index	Hallux angle [°]
6 to <10 years	Difference	−0.58	−0.20	−0.03	0.01	−0.00	−1.43
	(95%-CI)	(−0.99,−0.16)	(−0.35,−0.05)	(−0.03,−0.02)	(0.01,0.02)	(−0.02,0.02)	(−2.86,0.01)
	p	0.006	0.010	<0.001	<0.001	0.822	0.052
10 to <14 years	Difference	−0.04	−0.00	−0.02	0.01	0.03	−1.22
	(95%-CI)	(−0.38,0.29)	(−0.12,0.12)	(−0.03,−0.02)	(0.00,0.01)	(0.02,0.04)	(−2.37,−0.06)
	p	0.802	0.978	<0.001	0.025	<0.001	0.039
14 to 18 years	Difference	−0.63	0.00	−0.02	0.01	0.00	−1.16
	(95%-CI)	(−0.97,−0.28)	(−0.13,0.13)	(−0.03,−0.02)	(0.00,0.01)	(−0.01,0.02)	(−2.36,0.04)
	p	<0.001	0.981	<0.001	0.014	0.696	0.059
Main hypotheses _α(adj)__=__0.008_	p_interaction_	0.028	0.081	0.310	0.329	0.004	0.959
Hierarchical hypotheses _α__=__0.05_	p_barefoot *vs*. shod_^§^	(*0.001*)	0.204	<0.001	<0.001	*(0.010)*	0.001

§In parentheses p-value of group-effect modeled without significant interaction.

## Discussion

To better understand the effects of habitual barefoot locomotion on foot morphology during childhood and adolescence, this epidemiological study compared key measurements of foot characteristics between habitually barefoot and shod children and adolescents between 6 and 18 years of age. The main effects of growing up barefoot – compared to shod – were found for static arch index, pliability of the feet and hallux angles.

### Foot arch

One of the main findings of this study was the increased static arch height index in children and adolescents growing up barefoot. This is in accordance with lower incidences of flat feet reported for habitually barefoot children using static measures^4, 9, 11^. To our knowledge, only one study compared statically measured arch characteristics between habitually barefoot and shod adults and did not find a statistically significant difference^8^. The differences between their and our findings could lie in a different assessment method (navicular height and drop *vs*. sitting and standing static arch height), in the smaller sample size (255 *vs*. 810) or in the different populations examined (>18 years *vs*. ≤18 years). From other research, we know that there is a similar comparable development of the medial longitudinal arch in children between cultures and ethnicities^25^. Since we adapted our analysis for possible confounders such as sex, ethnicity, BMI and physical activity, our findings suggest that habitual footwear use influences the development of foot arch morphology in children.

The dynamically measured arch index differed only in the age group 10–14 years with higher values (=flatter arch) in the habitually shod cohort. Even though, in this age group the findings are analogous to our statically assessed foot arch, the results for the dynamically assessed foot arch differ in the age groups 6–10 and 14–18 years. There are two main theories which are frequently discussed in the etiology of reduced foot arch height^26^. One considers the strength of bones and ligaments, while the other discusses muscle strength^26^. Neither factor was assessed in our study, though one can only speculate about the underlying mechanism. There is evidence for altered biomechanics which are dependent on footwear^27, 28^, and a recent study investigated the activation of intrinsic foot muscles when running either barefoot or shod^29^. Kelly *et al*.^29^ showed an altered activation pattern for the flexor digitorum brevis and abductor hallucis muscles, which was dependent on the use of footwear. In addition to these intrinsic muscles, the tibialis posterior and flexor hallucis longus muscles have an impact on the foot arch as well. Another study^30^ found poor extensor muscle activity during the heel-contact phase in children with flexible flat feet. The possibility of altered muscle tone could also have resulted in the lifting of the medial longitudinal arch. In accordance with the “bone and ligaments” theory, a long-term altered muscle activation could also result in an adaptation of the bones and ligament of the foot^26^. This theory points to underlying mechanisms that might explain the differences between children who are habituated to barefoot or to shod walking.

However, this does not explain why differences were found in only one of the three age groups. There is a certain plasticity of the arch during childhood development which puberty seems to impact^31–33^. To our knowledge, there are no longitudinal studies investigating the effect of footwear on the development of the foot during this stage of development. Therefore, at this time, one can only speculate about the underlying mechanism affecting arch plasticity in the feet of habitually barefoot *vs*. shod children during puberty. It is known that fat distribution and ligamentous strength transform over time^32^, but hormonal changes (e.g. growth hormones and testosterone) also influence the bone and muscle growth during this phase^34^. Thus, it is plausible that the years between 10–14 is also important for foot arch development, but this idea needs further attention with a prospective investigation of foot development in this phase.

Another reason for the different findings can be found in the lack of comparability between static and dynamic arch measurements. In a pilot study, we found a high reliability for both measurements but a low correlation of both indices^19^. In this study, we have different viewpoints on the foot: direct static and indirect dynamic assessments. The dynamic arch index represents the area of the middle third of the pedobarographically measured footprint and can be influenced by skin thickness and plantar fat distribution. This would also be in accordance with the different confounding factors influencing dynamic arch index and static arch height index (dynamic: side, African ethnicity and BMI *vs*. static: side and sex) in this study (Fig. 2). Even though the findings for dynamic and static arch morphology is not congruent, we still think that having both assessments in our field study is a strength and will make the results more comparable to other studies. For future research, we would advise taking care when comparing results from dynamic and static arch measurements^19^.

Another large epidemiological study showed that the dynamically measured arch index appears to be stable from around 6 years of age in German children^15^. This is not the case for our cohorts. A prospective study design would be appropriate to better understand the development of the arch structure during childhood.

Taking all our data into consideration, one can conclude that arch morphology differs significantly between habitually barefoot and shod children and adolescents with flatter foot arches when growing up shod.

### Foot length and width

While controlled for sex, BMI, ethnicity and physical activity, we found longer feet in the age groups 6–10 and 14–18 years and wider feet in the younger participants (6–10 years) for habitually barefoot participants. There have been several attempts to compare shod and unshod feet of adults with conflicting evidence for foot length and limited evidence for an increased foot width in barefoot populations^4, 7, 8, 35^. Only one of the studies considered children, but did not distinguish age groups or give direct comparisons to shod counterparts^35^. None of these studies controlled for confounding variables such as BMI or ethnicity. D’Août and colleagues^8^ used two shod control groups – one from the same and one from a different ethnic background. This was not possible in our comparison since the rate of habitually shod children was too small in South Africa (<10%). Taking the conflicting evidence on this topic into consideration^4^, we can only speculate on possible mechanism underlying the observed effects, such as body weight, body size or genetic aspects which influence, for example, ligament laxity^35, 36^. Our findings are practically relevant for the development of footwear to ensure an accurate fit.

### Pliability

When comparing the pliability of the participants’ feet, we found significantly different pliability ratios in habitually shod children compared to their habitually barefoot counterparts. To our knowledge, this is the first study comparing the pliability of feet in a pediatric cohort. Nonetheless, our findings on differences in pliability are in agreement with another study investigating the pliability of shod and unshod feet of adults^6^. Kadambande and colleagues also showed that the differences in pliability were not caused by the intrinsic muscle activity. While it is not clear whether high or low pliability ratios are beneficial, the affected pliability is discussed as a cause of foot pathologies such as hallux valgus, hallux rigidus and pes planus^6, 18, 37^. Furthermore, it has been speculated that the pliability of the foot might impact running performance, especially in consideration of evidence which exists for a “spring-like function of the foot”^29, 38^. Further research is needed, however, to better understand the relationship between pliability, running performance and pathologies of the foot.

### Hallux valgus angle

The hallux valgus angle of habitually barefoot children in this study showed higher values in all age groups. This finding is surprising, having contrary findings in adult barefoot populations in mind^7, 39^. Footwear, especially constraining and high-heeled footwear, has been discussed as an extrinsic risk factor for the development of hallux valgus deviations^37, 40^. Perera *et al*. conclude in their review article that in the pathogenesis of hallux valgus several risk factors (amongst others: genetics, ligamentous laxity, pes planus and age) have to come together. As seen in this study, the habitually barefoot children tend to have a higher medial longitudinal arch which could be protective for the development of a hallux valgus^37^. However, there are conflicting findings with regard to the relation between pes planus and hallux valgus in children^37, 41^. As age is a separate risk factor, longitudinal data would be needed to better understand the development of the hallux and why the incidence of hallux valgus deviations is higher in habitually shod adult populations^7, 39^. Whether the differences found are or will be clinically relevant still has to be determined.

Another explanation for our findings could lie in the fact that, while being defined as habitually barefoot, the children in our study could have used footwear infrequently. Even though not assessed in this study, it is possible that the footwear (such as part of the school uniform in secondary schools) of the habitually barefoot children was more constrictive than the footwear of the habitually shod children. There has been an ongoing debate on the correct shoe size for several years in Europe^40^ and there is at least some awareness of the importance of correctly-fitting footwear^42^.

Furthermore, the reader shall be reminded that the hallux angle was measured pedobarographically in the field. There have been other studies using the method^4, 7^, but radiographic measurement is still the gold standard in the assessment of the hallux.

### Limitations

One of the strengths of this study is at the same time a limitation. After an initial systematic screening of the literature, we found that studies reporting on barefoot populations do not use a common definition of the term “habitually barefoot”^4^. Therefore, the decision was made to use a barefoot questionnaire. To the authors’ knowledge, only D’Août and colleagues^8^ used an unpublished questionnaire to determine habitually barefoot study participants. The test-retest reliability was good (ICC = 0.691), but a proper validation has not yet been conducted. This would require a direct observation of the children for a longer period. For further research purposes, we published the questionnaire prior to the study together with the study protocol^18^. We acknowledge that this is a new approach and that the comparability of studies investigating habitually barefoot populations has not yet been determined. Nonetheless, we hope that it helps other researchers and will be used and modified for a consensus on the term “habitually barefoot”. Due to the missing validation, we decided to not further investigate the impact of the barefoot questionnaire score for subgroup analysis but for the inclusion of our habitually barefoot participants. We look forward to future discussion of this aspect.

Another limitation is with regard to data collection. Since we collected our data in school settings of urban and rural areas of South Africa and Germany, we had to use less specialized equipment than would normally be used in a gait laboratory or orthopedic clinic. Lastly, during dynamic foot measurement, the participants’ walking velocity was self-selected and not monitored and therefore might have influenced the comparability.

### Generalizability

The generalizability of our data needs some critical considerations. We used an epidemiological approach and aimed to recruit participants from similar ethnic backgrounds^18^. In other research, the ethnic backgrounds were not always taken into consideration, which is fundamental due to significant foot morphological differences between ethnicities^14^. In this study mainly children and adolescents with a Caucasian ethnicity (>90%) were tested. Furthermore, we adapted our statistical analysis for ethnicity as a confounding factor. All of this contributed in securing a comparison of our independent variable: growing up habitually barefoot or shod. Nonetheless, care should be given when comparing our findings with other ethnic or adult populations.

## Conclusion

Despite the increasing interest in barefoot locomotion, a habit which has been part of human evolution for millions of years, the evidence is small for its long-term effects on foot characteristics. This study helps to understand what consequences can be found for foot development when growing-up barefoot *vs*. shod. It shows that permanent footwear use may play an important role in childhood foot development and might actually be beneficial for the development of the foot arch. Future research should focus on a harmonization of the definition for the term “habitual barefootness” as well as for the clinical and practical consequences of our findings.

### Availability of data and materials

All data generated or analysed during this study are included in this published article and its supplementary tables and figures, or is available upon request.

### Ethics approval and consent to participate

Ethics approval has been obtained from the ethics committee of the medical association Hamburg (protocol number PV4971) and Stellenbosch University ethics committee (protocol number HS1153/2014). Written informed parental consent and the child’s assent to participate was obtained prior to participation.

## Electronic supplementary material

Table S1
