# Supplementary material for: Growing-up (habitually) barefoot influences the development of foot and arch morphology in children and adolescents
Source: Sci Rep. 2017 Aug 14;7:8079. doi: 10.1038/s41598-017-07868-4 (PMC5556098; doi:10.1038/s41598-017-07868-4)
Supplement: Supplementary file 1 — Table S1 [file 41598_2017_7868_MOESM1_ESM.doc]

**Growing-up (habitually) barefoot influences the development of foot and arch morphology in children and adolescents**

Karsten Hollander, Johanna Elsabe de Villiers, Susanne Sehner, Karl Wegscheider, Klaus-Michael Braumann, Ranel Venter, Astrid Zech

**Supplemental Table 1.** Estimated means of habitually barefoot and shod children by age in stages of development.

| **Estimated marginal means of habitually barefoot *vs.* habitually shod children** | | Foot length  [cm] | Foot width  [cm] | Static arch height  index | Pliability Ratio | Dynamic arch  index | Hallux angle  [°] |
| --- | --- | --- | --- | --- | --- | --- | --- |
|  | | | | | | | |
| **6 to < 10 years** |  |  |  |  |  |  |  |
| Habitually barefoot | Est. means | 21.34 | 8.41 | 0.28 | 1.05 | 0.18 | 0.76 |
|  | (95%-CI) | (21.03,21.64) | (8.30,8.52) | (0.27,0.28) | (1.04,1.05) | (0.17,0.19) | (-0.28,1.80) |
| Habitually shod | Est. means | 20.76 | 8.21 | 0.25 | 1.06 | 0.18 | -0.66 |
|  | (95%-CI) | (20.46,21.06) | (8.10,8.32) | (0.25,0.25) | (1.05,1.06) | (0.17,0.19) | (-1.73,0.40) |
| **10 to < 14 years** |  |  |  |  |  |  |  |
| Habitually barefoot | Est. means | 23.63 | 8.99 | 0.27 | 1.04 | 0.17 | 3.06 |
|  | (95%-CI) | (23.39,23.87) | (8.90,9.08) | (0.26,0.27) | (1.04,1.05) | (0.16,0.17) | (2.24,3.88) |
| Habitually shod | Est. means | 23.59 | 8.99 | 0.24 | 1.05 | 0.19 | 1.84 |
|  | (95%-CI) | (23.36,23.81) | (8.90,9.07) | (0.24,0.25) | (1.05,1.05) | (0.19,0.20) | (1.07,2.61) |
| **14 to 18 years** |  |  |  |  |  |  |  |
| Habitually barefoot | Est. means | 25.24 | 9.31 | 0.27 | 1.03 | 0.17 | 4.01 |
|  | (95%-CI) | (24.96,25.52) | (9.21,9.41) | (0.26,0.27) | (1.03,1.04) | (0.16,0.18) | (3.05,4.97) |
| Habitually shod | Est. means | 24.61 | 9.31 | 0.24 | 1.04 | 0.17 | 2.85 |
|  | (95%-CI) | (24.39,24.83) | (9.23,9.39) | (0.24,0.25) | (1.04,1.05) | (0.16,0.18) | (2.09,3.62) |
